# Cephalometric Evaluation of Mouth-Breathing Patients: A Retrospective Study Using Sassouni, Down, and Steiner Analyses

**DOI:** 10.7759/cureus.97925

**Published:** 2025-11-27

**Authors:** Bhawana Kumari, Rajeev K Singh, Richa Khanna

**Affiliations:** 1 Paediatric and Preventive Dentistry, King George’s Medical University, Lucknow, IND

**Keywords:** facial asymmetry, lateral cephalometry, malocclusion of teeth, mouth breathing, young child

## Abstract

Background

Mouth breathing is a prevalent condition in children and is often associated with altered craniofacial development. Cephalometric analysis is a key diagnostic tool used to evaluate these changes. However, different cephalometric analyses may yield inconsistent diagnostic outcomes, potentially affecting treatment decisions. This study aimed to evaluate and compare skeletal anteroposterior relationships, maxillary and mandibular positions, and growth direction in mouth-breathing children using Sassouni, Down, and Steiner analyses.

Methodology

This retrospective, cross-sectional study included 88 lateral cephalometric radiographs of mouth-breathing children aged 6-14 years. Cephalograms were analyzed independently using the Sassouni, Down, and Steiner methods. Parameters assessed included skeletal classification, maxillary and mandibular positions, and facial growth direction. Statistical analyses were performed using paired t-tests, chi-square tests, and kappa statistics to evaluate inter-method variability and significance.

Results

Significant differences were found among the three analyses in all evaluated parameters. The Sassouni method identified more Class II skeletal patterns and normal maxillary positions compared to the Steiner method, which showed higher rates of retrognathic jaws. Down’s analysis indicated a greater proportion of normal growth direction than Sassouni’s. All differences were statistically significant (p < 0.05), highlighting substantial diagnostic variability.

Conclusions

These findings underscore the diagnostic inconsistency among commonly used cephalometric analyses in assessing mouth-breathing children. These variations have important clinical implications, emphasizing the need for a comprehensive diagnostic approach that integrates multiple analyses and clinical assessments to guide accurate treatment planning.

## Introduction

The oral cavity plays a critical role in fundamental physiological functions such as respiration, mastication, speech, and overall craniofacial development [1]. These functions are intricately coordinated, and any disruption, particularly in breathing patterns, can significantly affect the morphology and growth of the craniofacial complex [2,3]. Among these disruptions, mouth breathing stands out as a common and clinically relevant condition, especially in the pediatric population [4].

Mouth breathing (also termed as oral breathing) is defined as a habitual pattern of inhaling and exhaling through the mouth instead of the nose, typically persisting for more than six months [5,6]. This altered respiratory pattern is often secondary to upper airway obstructions such as adenoidal or tonsillar hypertrophy, deviated nasal septum, allergic rhinitis, or other anatomical or functional causes [7]. In many cases, mouth breathing serves as an adaptive mechanism to compromised nasal airflow. However, when persistent, it can lead to systemic, dentofacial, and postural consequences, particularly during periods of active craniofacial growth [8].

Numerous studies have highlighted the potential impact of chronic mouth breathing on facial skeletal development. Reported changes include increased anterior facial height, retrognathic mandible, narrow maxillary arch, lip incompetence, and a high incidence of malocclusion such as Class II relationships, open bite, and posterior crossbite [9,10]. These alterations are often collectively referred to as “adenoid facies” or “long face syndrome” [11,12]. Functional adaptations such as lowered tongue posture, altered head and neck position, and muscular imbalance further exacerbate these morphological changes [13]. While Moss and Salentijn’s functional matrix theory provides a conceptual framework suggesting that normal nasal breathing supports harmonious craniofacial development, the exact nature of the association between mouth breathing and skeletal alterations remains a subject of debate [2].

The diagnostic evaluation of these skeletal and dental changes is often conducted through cephalometric analysis, a standardized radiographic technique used to assess craniofacial morphology [14]. Among the many cephalometric analyses available, the Sassouni, Down, and Steiner methods are widely recognized for evaluating skeletal anteroposterior relationships, maxillary and mandibular positions, and growth direction. However, variations exist between these analyses in terms of reference points, measurements, and diagnostic interpretations, which may influence clinical decision-making [15].

Given the ongoing controversy regarding the morphological consequences of mouth breathing and the diagnostic inconsistency among cephalometric analyses, this study aims to evaluate and compare the cephalometric findings in mouth-breathing patients using the Sassouni, Down, and Steiner analyses. Specifically, the study investigates skeletal anteroposterior relationships, maxillary and mandibular positions, and growth direction to determine if significant variations exist among these methods.

## Materials and methods

Study design and setting

This retrospective, cross-sectional, analytical study was conducted in the Department of Paediatric and Preventive Dentistry, Faculty of Dental Sciences, King George’s Medical University (KGMU), Lucknow, Uttar Pradesh, India. The study aimed to evaluate and compare cephalometric characteristics of children diagnosed with chronic mouth breathing using three established methods, namely, Sassouni, Down, and Steiner analyses [15]. Ethical approval for the study was obtained from the Institutional Ethical Committee of KGMU (approval number: 34th PGTSC-IIA/P17). All procedures adhered to the principles outlined in the Declaration of Helsinki, with strict confidentiality maintained for patient records and radiographs [16].

Study population

The study included lateral cephalometric radiographs (cephalograms) of patients aged between 6 and 14 years who had a documented clinical diagnosis of chronic mouth breathing. These cephalograms were retrospectively collected from the radiographic and clinical archives of the department.

Diagnostic criteria for mouth breathing

Diagnosis of chronic mouth breathing was based on both detailed patient history and standardized clinical evaluation [17]. Clinical indicators included habitual open-mouth posture at rest, lip incompetence, dry lips, anterior open bite, and a history of nasal obstruction or snoring. Functional tests such as the mirror test (fogging test) and water retention test were used to confirm mouth-breathing tendency. Children demonstrating these features for more than six months were classified as chronic mouth breathers. Though objective tests such as rhinomanometry or nasoendoscopy could not be applied retrospectively, the diagnostic approach was consistent with established pediatric dental protocols.

Inclusion and exclusion criteria

High-quality, undistorted cephalograms of patients aged between 6 and 14 years at the time of radiographic acquisition with a confirmed diagnosis of chronic mouth breathing and with the availability of complete clinical records were included. Cephalograms of syndromic conditions affecting craniofacial development, such as cleft lip/palate or craniosynostosis, as well as those who had undergone prior orthodontic treatment, myofunctional therapy, or craniofacial surgery, were excluded. Cephalograms taken during periods of temporary mouth breathing due to acute infections and records with missing or incomplete clinical data were also excluded.

Sample size estimation

The sample size (n = 88) was estimated based on findings from a preliminary pilot study involving 20 subjects, which demonstrated approximately 66% diagnostic agreement between the Sassouni and Steiner analyses. Considering a statistical power of 90%, a significance level (α) of 0.05, and an allowable error of 15%, the minimum required sample size was calculated using the formula for comparing proportions. Accordingly, 88 cephalograms were included in the final analysis.

Data collection procedure

Two calibrated investigators independently screened the selected cephalograms and performed tracings according to the Sassouni, Down, and Steiner analyses. Radiographs were anonymized before assessment. Standardized tracing protocols were followed to ensure consistency. To evaluate inter-examiner reliability, 20% of the cephalograms were re-traced randomly, and kappa statistics were used to determine agreement levels.

Cephalometric parameters evaluated

For each cephalogram, skeletal anteroposterior relationships (Class I, II, III), sagittal positioning of the maxilla and mandible, and direction of facial growth (horizontal, vertical, or average) were recorded according to each analysis method. Angular and linear measurements were taken as per established norms for Sassouni, Down, and Steiner analyses.

Statistical analysis

The collected data were compiled and organized using SPSS Statistics for Windows, Version 23.0 (released 2015, IBM Corp., Armonk, NY, USA). Continuous variables were expressed as mean ± SD, and categorical data as frequencies and percentages. The paired t-test and Wilcoxon signed-rank test were used for inter-method comparison, while the chi-square test was used to assess associations between categorical parameters. Inter-method and inter-examiner agreement were evaluated using Cohen’s kappa statistics. A p-value <0.05 was considered statistically significant. Descriptive data were also stratified by age and gender to assess distributional patterns. Although multivariate adjustment was not performed (as the aim was inter-method comparison rather than causal inference), potential confounders were recognized and discussed accordingly.

## Results

A total of 88 lateral cephalograms of mouth-breathing children aged 6-14 years (mean age = 11.0 ± 2.07 years) were analyzed, with an equal gender distribution (44 males and 44 females). Cephalometric findings revealed a high prevalence of retrognathic mandibles, vertical growth tendencies, and skeletal discrepancies in this population. Comparative evaluation among the Sassouni, Down, and Steiner analyses demonstrated statistically significant differences in skeletal classification, jaw positioning, and growth direction, highlighting inconsistencies in diagnostic outcomes across methods.

Kappa statistics were calculated to assess the level of agreement between the three cephalometric analyses and between the two examiners. Inter-examiner reliability was found to be substantial (κ = 0.78), indicating consistent tracing and classification across evaluators. However, inter-method agreement ranged from fair to moderate, with κ values of 0.34 (Sassouni vs. Steiner), 0.42 (Sassouni vs. Down), and 0.39 (Down vs. Steiner), suggesting notable diagnostic variability across methods.

Comparison of skeletal anteroposterior relationships

The Sassouni analysis identified a Class I skeletal pattern in 48 (54.5%) cases, while Down’s analysis (based on facial angle) reported a higher prevalence of Class I in 64 (72.7%) cases. Conversely, Class II relationships were observed in 37 (42.0%) cases with Sassouni compared to 23 (26.1%) with Down’s analysis. The difference between the two analyses was statistically significant (χ² = 6.55, p = 0.038), suggesting variation in skeletal classification based on the method used (Table 1).

**Table 1:** Comparison of skeletal anteroposterior/facial angle between Sassouni analysis (skeletal anteroposterior) and Down analysis (facial angle).

Skeletal anteroposterior/facial angle	Sassouni analysis (Skeletal anteroposterior)		Down analysis (facial angle)		Chi-square	Significance
Class I	48	54.50%	64	72.70%	6.55	P = 0.038
Class II	37	42.00%	23	26.10%		
Class III	3	3.40%	1	1.10%		

Comparison of maxillary position

A notable discrepancy was observed between Sassouni and Steiner analyses in the evaluation of maxillary position. Sassouni classified 68 (77.3%) cases as having a normal maxilla, while Steiner’s sella-nasion-A (SNA) angle indicated normal positioning in only 15 (17.0%) cases. Retrognathic maxilla was detected in 16 (18.2%) cases by Sassouni and in 48 (54.5%) cases by Steiner analysis. This difference was statistically significant (χ² = 65.05, p < 0.001), indicating substantial variation in diagnosis based on analysis type (Table 2).

**Table 2:** Comparison of maxillary position/SNA between Sassouni analysis (skeletal anteroposterior) and Steiner analysis (SNA). SNA = sella-nasion-A point

Maxillary position/SNA	Sassouni analysis (maxillary position)		Steiner analysis (SNA)		Chi-square	Significance
Normal	68	77.30%	15	17.00%	65.05	P < 0.001
Retrognathic	16	18.20%	48	54.50%		
Prognathic	4	4.50%	25	28.40%		

Comparison of mandibular position

Assessment of mandibular positioning also differed between the two analyses. Sassouni identified 63 (71.6%) cases as having a retrognathic mandible, whereas Steiner’s sella-nasion-B (SNB) angle indicated retrognathism in 81 (92.0%) cases. Only two (2.3%) cases were labelled as normal by Steiner analysis, compared to 23 (26.1%) by Sassouni. The difference was statistically significant (χ² = 21.17, p < 0.001) (Table 3).

**Table 3:** Comparison of mandibular position/SNB between Sassouni analysis (mandibular position) and Steiner analysis (SNB). SNB = sella-nasion-B point

Mandibular position/SNB	Sassouni analysis (mandibular position)		Steiner analysis (SNB)		Chi-square	Significance
Normal	23	26.10%	2	2.30%	21.17	P < 0.001
Retrognathic	63	71.60%	81	92.00%		
Prognathic	2	2.30%	5	5.70%		

Comparison of growth direction

The growth direction evaluation showed significant disagreement between Sassouni and Down’s Y-axis analysis. Sassouni reported a normal growth pattern in 42 (47.7%) cases, while Down’s Y-axis indicated normal growth in 72 (81.8%) cases. Vertical growth patterns were observed in 26 (29.5%) cases by Sassouni and only nine (10.2%) by Down’s analysis. The difference was statistically significant (χ² = 22.41, p < 0.001), reflecting inconsistencies between methods in identifying vertical growers (Table 4).

**Table 4:** Comparison of growth direction/Y-axis between Sassouni analysis (growth direction) and Down analysis (Y-axis).

Growth direction/Y-axis	Sassouni analysis (growth direction)		Down analysis (Y-axis)		Chi-square	Significance
Normal	42	47.70%	72	81.80%	22.41	P < 0.001
Horizontal grower	20	22.70%	7	8.00%		
Vertical grower	26	29.50%	9	10.20%		

## Discussion

This study demonstrated notable variability in the cephalometric assessment of mouth-breathing children depending on the analysis method used. Sassouni’s analysis identified a higher proportion of Class II skeletal patterns in 37 (42.0%) cases, compared to Down’s analysis, which reported more cases as Class I in 64 (72.7%) cases. In evaluating maxillary position, Sassouni classified 68 (77.3%) cases as normal, whereas Steiner’s analysis identified only 15 (17.0%) cases as normal, with a much higher prevalence of retrognathic maxilla in 48 (54.5%) cases and prognathic maxilla in 25 (28.4%) cases. Similarly, for mandibular position, Steiner’s analysis indicated retrognathism in 81 (92.0%) cases, significantly higher than Sassouni’s 63 (71.6%) cases. When assessing growth direction, Down’s Y-axis analysis suggested a normal growth pattern in 72 (81.8%) cases, while Sassouni found normal growth in 42 (47.7%) cases, reporting more cases with vertical growth tendencies in 26 (29.5%) cases. These differences were statistically significant across all parameters, indicating that diagnostic interpretations vary substantially between cephalometric methods.

The findings of this study fit within the broad literature related to mouth breathing and facial growth. Prior cephalometric studies consistently report that mouth-breathing children have a tendency toward a more retrusive mandible, increased lower facial height, and Class II skeletal patterns. Munoz et al. [6] found mouth breathers had significantly lower SNB (retrognathic mandible) and a more steeply inclined mandibular plane angle than nasal breathers. Malhotra et al. [1] also observed increased total and lower anterior face heights and higher sella-nasion to gonion-gnathion (SN-GoGn) angles in mouth breathers. A recent meta-analysis by Zhao et al. [18] similarly reported that mouth-breathing children have reduced SNA/SNB (maxillary and mandibular retrusion), increased point A-nasion-point B (ANB), and steeper vertical angles (e.g., SN-GoGn). In line with these studies, our sample participants overall demonstrated the classic “long-face” and Class II tendencies described in the literature.

However, no previous work has directly examined how different cephalometric analyses compare in this population. In contrast to our results, Kuyl et al. [19] found that Steiner and Sassouni’s analyses produced essentially the same sagittal classification in adult patients. The study suggested only minor differences in sagittal profile scores between Sassouni and Steiner analyses. By contrast, we observed substantial disagreements in skeletal classification for mouth-breathing children. This discrepancy may reflect the more variable craniofacial morphology of mouth breathers or sample differences owing to growth stages. The findings of the study, in any case, contribute to the literature substantially by showing that, even in a cohort predisposed to vertical, Class II features, the choice of analysis markedly alters the diagnosis.

The observed variability among cephalometric analyses holds important implications for orthodontic diagnosis and treatment planning in mouth-breathing children. When sagittal discrepancies or growth patterns are classified differently depending on the analytic method, clinicians may arrive at contrasting treatment decisions. These diagnostic variations underscore the need for caution when relying on a single cephalometric analysis. As highlighted in the literature, cephalometric findings must always be interpreted in the broader clinical context, as factors such as soft tissue profile and dentoalveolar compensations may conceal underlying skeletal discrepancies [20]. Clinicians are, therefore, advised to integrate multiple diagnostic indicators, such as ANB angle, Wits appraisal, and overall facial harmony, alongside a comprehensive clinical and functional assessment, including evaluation of airway and respiratory patterns. In complex or borderline cases, advanced imaging modalities such as cone-beam CT (CBCT) or MRI may provide additional clarity to guide effective treatment planning.

The discrepancies observed between the cephalometric analyses in the present study can be attributed to differences in landmarks, reference planes, and measurement techniques utilized by different analyses. Steiner analysis relies on the SN plane and ANB angle, assuming consistent cranial base landmarks. Down’s analysis typically uses the Frankfort horizontal and vertical references, such as nasion or pterygoid point, while Sassouni’s method incorporates multiple planes and their intersections, integrating both sagittal and vertical assessments. Variations in identifying anatomical points, such as nasion or gonion, or differences in head positioning can significantly influence angular measurements. Moreover, studies have shown only moderate agreement between soft-tissue profiles and Steiner-based classifications, reflecting inherent variability in these metrics [21].

All three methods are based on two-dimensional cephalograms, which cannot account for transverse asymmetries or out-of-plane head rotations [22,23]. This is particularly relevant for mouth-breathing patients, who often exhibit altered head posture. Such limitations can distort spatial relationships and reduce diagnostic accuracy. While three-dimensional imaging, such as CBCT, was beyond the scope of this retrospective study, it offers more precise landmark identification and eliminates superimposition errors [24]. Overall, methodological differences in landmark selection, reference planes, and imaging modality likely contributed to the diagnostic inconsistencies observed in this study.

The present study has several strengths, including being one of the first to systematically compare three widely used cephalometric analyses, namely, Sassouni, Down, and Steiner, within the same cohort of mouth-breathing children. Applying multiple methods to a single sample allowed for direct assessment of inter-method variability, and the use of calibrated examiners with inter-rater reliability checks ensured consistency. The adequate sample size and broad age range (6-14 years) captured a critical period of craniofacial development. However, the study also has limitations. Its retrospective design limits causal inference and may introduce selection bias, as participants were pre-selected for cephalometric evaluation. The absence of a control group of nasal breathers restricts the generalizability of findings. Reliance on two-dimensional lateral cephalograms prevents assessment of transverse dimensions and airway space, and potential inaccuracies in landmark identification could influence measurements. Moreover, only three analyses were included, and other methods, such as Ricketts or McNamara, might have produced different results. Future studies can plan to assess the diagnostic accuracy of these analyses and help in identifying and categorizing preferred analyses in different population groups.

## Conclusions

This study highlights that the choice of cephalometric analysis substantially affects the perceived craniofacial diagnosis in mouth-breathing children. Significant discrepancy between Sassouni, Down, and Steiner classifications suggests that clinicians should not rely on a single metric. Instead, multiple analyses and holistic evaluation (including soft-tissue profile and functional assessment) should form the diagnosis and planning. Our findings underscore the importance of interpreting cephalometric data within its clinical context and suggest that future use of three-dimensional imaging or standardized protocols could improve diagnostic consistency.
